# Local light signaling at the leaf tip drives remote differential petiole growth through auxin-gibberellin dynamics

**DOI:** 10.1016/j.cub.2022.11.045

**Published:** 2023-01-09

**Authors:** Jesse J. Küpers, Basten L. Snoek, Lisa Oskam, Chrysoula K. Pantazopoulou, Sanne E.A. Matton, Emilie Reinen, Che-Yang Liao, Eline D.C. Eggermont, Harold Weekamp, Muthanna Biddanda-Devaiah, Wouter Kohlen, Dolf Weijers, Ronald Pierik

**Affiliations:** 1 Plant-Environment Signaling, Department of Biology, Utrecht University, Padualaan 8, 3584 CH Utrecht, the Netherlands; 2 Theoretical Biology and Bioinformatics, Department of Biology, Utrecht University, Padualaan 8, 3584 CH Utrecht, the Netherlands; 3 Laboratory of Biochemistry, Wageningen University, Stippeneng 4, 6708 WE Wageningen, the Netherlands; 4 Laboratory for Molecular Biology, Wageningen University, Droevendaalsesteeg 1, 6708 PB Wageningen, the Netherlands

**Keywords:** phytochrome, shade avoidance, neighbor detection, hyponasty, leaf movement, auxin, gibberellin, RNA sequencing, C3PO, confocal microscopy

## Abstract

Although plants are immobile, many of their organs are flexible to move in response to environmental cues. In dense vegetation, plants detect neighbors through far-red light perception with their leaf tip. They respond remotely, with asymmetrical growth between the abaxial and adaxial sides of the leafstalk, the petiole. This results in upward movement that brings the leaf blades into better lit zones of the canopy. The plant hormone auxin is required for this response, but it is not understood how non-differential leaf tip-derived auxin can remotely regulate movement. Here, we show that remote signaling of far-red light promotes auxin accumulation in the abaxial petiole. This local auxin accumulation is facilitated by reinforcing an intrinsic directionality of the auxin transport protein PIN3 on the petiole endodermis, as visualized with a PIN3-GFP line. Using an auxin biosensor, we show that auxin accumulates in all cell layers from endodermis to epidermis in the abaxial petiole, upon far-red light signaling in the remote leaf tip. In the petiole, auxin elicits a response to both auxin itself as well as a second growth promoter; gibberellin. We show that this dual regulation is necessary for hyponastic leaf movement in response to light. Our data indicate that gibberellin is required to permit cell growth, whereas differential auxin accumulation determines which cells can grow. Our results reveal how plants can spatially relay information about neighbor proximity from their sensory leaf tips to the petiole base, thus driving adaptive growth.

## Highlights

• Temporal transcriptome patterns in response to shade cues in leaf segments
• Tissue-specific quantification of auxin reporter in petioles
• Auxin and Gibberelin interactively regulate leaf movement
• Mechanisms underpinning physical separation of light signaling and response

## Teaser

Küpers et al. identify how plant neighbor detection via light signals at the leaf tip, drives upward leaf movement. They show that the hormone auxin is transported from the leaf tip to the abaxial side of the base, where it promotes cell growth. These findings reveal how plants spatially distribute information of plant proximity.

## Introduction

In dense vegetation, plants adapt their growth to actively compete for light with their neighbors. However, light distribution in vegetation is heterogeneous, and different plant parts therefore receive different light intensities and density cues.^1^ Plants use intricate signal transfer mechanisms between plant parts to respond adequately to this heterogeneous information,^2^^,^^3^^,^^4^ but these mechanisms remain poorly understood. In *Arabidopsis*, adaptive shade avoidance responses include hypocotyl elongation in seedlings and petiole elongation and upward leaf movement (hyponasty) in adult plants.^5^ Although evolutionarily adaptive, shade avoidance responses reduce productivity of dense monocultures.^1^^,^^6^^,^^7^ To accurately evaluate the competitive threat in their environment, plants use phytochrome (phy) photoreceptors to monitor red (R) to far-R (FR) light ratio (R/FR).^8^ In shade, the R/FR ratio decreases due to specific R light absorption by leaves to power photosynthesis. However, even before actual shading occurs, reflected FR-enriched light from neighboring leaves reduces the R/FR and provides an early neighbor proximity signal that precedes light competition.^9^ FR-enriched light induces tissue-specific growth responses in *Arabidopsis* leaves depending on the site of perception.^10^^,^^11^ FR enrichment at the petiole locally stimulates petiole elongation while FR enrichment at the leaf tip (FRtip) induces petiole hyponasty. The spatial separation between FR-induced petiole elongation and hyponasty allows the plant to optimally adjust its growth to either self-shading or neighbor competition.^11^ In FRtip-induced petiole hyponasty, there is spatial separation between the sensory leaf tip and the responding petiole base.^10^^,^^11^ Moreover, petiole hyponasty requires differential cell elongation between the abaxial (bottom) and adaxial (top) petiole sides.^12^ This hyponastic growth response thus provides a study system to unravel how remote light signaling regulates distal and differential growth without local light signaling in the growing tissue. We previously established that FRtip-induced petiole hyponasty occurs via local phyB inactivation in the leaf tip, which activates the PHYTOCHROME INTERACTING FACTOR (PIF) bHLH transcription factors.^10^^,^^11^ Active PIFs enhance the expression of *YUCCA* (*YUC*) genes that encode the YUC enzymes required for auxin biosynthesis.^13^^,^^14^^,^^15^ The auxin that is produced in the leaf tip subsequently stimulates petiole hyponasty. A similar regulatory network also drives seedling hypocotyl elongation upon FR enrichment in the cotyledons.^16^^,^^17^

So far, it remained unclear how the auxin signal from the remote leaf tip directs differential growth and petiole hyponasty, if this involves the build-up of an auxin gradient, in which tissues this would occur, and if other messengers are involved. Using tissue-specific time series RNA sequencing, we show that neighbor detection in the leaf tip induces unique transcript profiles in the leaf tip, the abaxial petiole, and the adaxial petiole. Leaf-tip-derived auxin is specifically transported toward the abaxial petiole to locally enhance gene expression and ultimately cell elongation. Besides auxin, we identify roles for gibberellin (GA) and PIFs in the responding petiole and suggest petiole side-specific signaling via members of the growth-promoting BRASSINAZOLE RESISTANT 1 (BZR1)—AUXIN RESPONSE FACTOR 6 (ARF6)—PIF4/DELLA (BAP/D) transcription factor module. This study reveals how plants use targeted long-distance auxin signaling to adapt their growth to competitive environments.

## Results

### Characterizing the kinetics and localization of FRtip-induced hyponasty and gene expression

**Figure 1:**
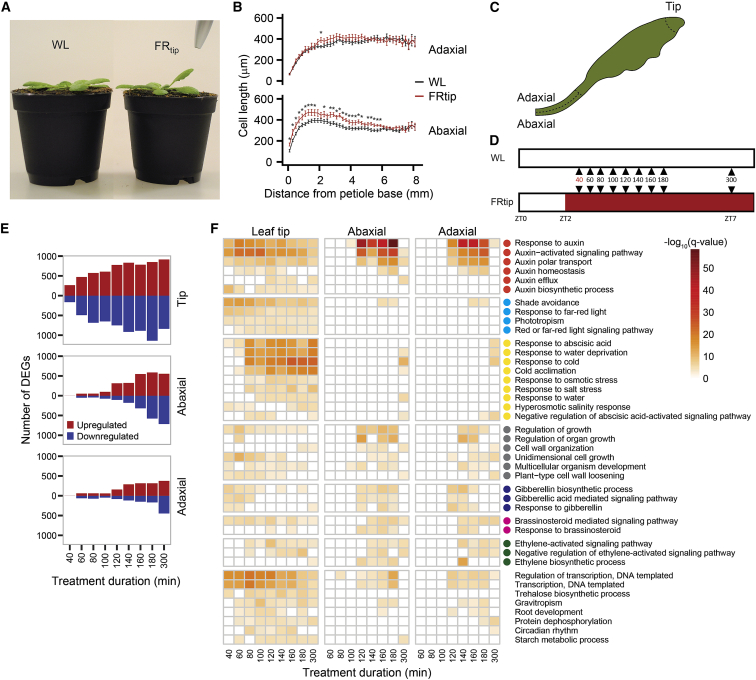
Neighbor detection in the leaf tip induces petiole hyponasty and transcriptional reprogramming in the petiole (A) Adult Col-0 phenotype after 24-h light treatments. (B) Epidermal cell length measured along the abaxial and adaxial petiole after 24 h light treatments (n = 12, WL; 15, FRtip, ^∗^p < 0.05, two-sided t test, data represent mean ± SEM). (C and D) Schematic representations of harvested material (C, dotted lines identify the harvested sections in leaf tip and petiole base) and harvest time points (D) for RNA sequencing. At the 40 min time point, only leaf tip material was analyzed. (E) Number of DEGs in FRtip compared with WL, calculated per time point and per tissue. DEGs were called when p < 0.01 and log_2_FC > 0.3 (upregulated; red) or log_2_FC < −0.3 (downregulated; blue). (F) Heatmap showing −log_10_(q value) of gene ontology (GO) terms identified per time point and per tissue based on upregulated DEGs defined in (E). Colored circles represent the following defined major biological processes: red, auxin distribution and signaling; cyan, light signaling; yellow, abscisic acid signaling; gray, cell and organ growth; blue, gibberellin biosynthesis and signaling; magenta, brassinosteroid signaling; green, ethylene biosynthesis and signaling. In FRtip, the R/FR is locally reduced from 2.0 to 0.05. See also Figures S1 and S2, Video S1, and Data S1.

Neighbor detection through FRtip induces visible petiole hyponasty ∼4 h after the treatment starts, slightly slower than in whole-plant supplemental FR exposure (Figures 1A and S1A; Video S1). To identify the responding cells, we measured epidermal cell length along the hyponastic petiole after 24 h of treatment and found that FRtip specifically enhances epidermal cell elongation in the proximal two-thirds of the abaxial petiole (Figure 1B). Considering the previously identified important role of auxin in FRtip-induced hyponasty, we studied auxin-responsive gene expression in the petiole upon FRtip. Indeed, the auxin-responsive *IAA29* and *ACS4* transcripts were induced in the proximal petiole within 100 min of FRtip while the shade marker transcript *PIL1* was unaffected in the non-FR-exposed petiole (Figure S1B). To better understand the spatial regulation of differential gene expression and petiole growth by FR signaling in the leaf tip, we separately harvested the leaf tip and the separated abaxial and adaxial sides of the responding proximal two-thirds of the petiole in white light (WL) and FRtip (Figure 1C). To capture early transcriptional responses, we harvested at 20 min intervals ranging from 60 min (40 min for the leaf tip) to 180 min of treatment as well as at a 300 min time point (Figure 1D).

Video S1. Dynamics of FRtip-induced leaf movement, related to Figures 1 and S1A Treatment duration in hours is indicated by the timer. Similar-sized leaves in the WL (left) and FRtip (right) treatments are indicated with an orange dot.

### Neighbor detection at the leaf tip induces local and remote, tissue-specific transcriptome changes

Reads were annotated to the TAIR10 genome and DESeq2-normalized read counts^18^ were used for principal coordinate analysis (PCoA) between all samples. We found clear PCoA separation between samples for time point and tissue type (Figure S1C). PCoA per tissue and differentially expressed gene (DEG) analysis per time point per tissue revealed consistent treatment effects in the leaf tip that only appeared at later time points in the petiole (Figures 1E and S1D; Data S1). To allow for quick analysis of individual transcripts, we made an easy-to-use Shiny application (https://kupersetal.shinyapps.io/Shiny/) that provides the FRtip-induced log_2_FC and significance range per tissue per time point, and the mean read counts per sample upon entry of an AGI locus found in the transcriptome.

### Neighbor detection at the leaf tip induces tissue-specific hormone response and biosynthesis

Gene ontology (GO) analysis for biological processes on upregulated DEGs revealed early enrichment of auxin and light quality-related GO terms in the leaf tip followed by enrichment of abscisic acid (ABA)-related GO terms (Figure 1F). As expected, in the petiole, light quality-related GO terms were largely absent. Here, auxin response terms were enriched from 100 to 180 min but dampened toward 300 min. This temporal GO enrichment pattern was similar for growth, brassinosteroid (BR) and ethylene response as well as GA biosynthesis and response (Figure 1F). Similar to the leaf tip, there was an enrichment of ABA-related GO terms in the petiole after the auxin response GO terms had passed peak significance. The ubiquitous overrepresentation of auxin signaling was confirmed when we analyzed expression of all genes comprising the GO category GO:0009733 “response to auxin” (Figure S2A). Analysis of these genes revealed shared but also time- and tissue-specific expression of many auxin-responsive genes. For example, regarding *SMALL AUXIN UPREGULATED* (*SAUR*) transcripts, *SAUR19-24* were induced in all tissues, while *SAUR25-29* and *SAUR62-68* were predominantly induced in the petiole (Figure S2A).

As we found GO enrichment for several hormone-related processes, we investigated hormone biosynthesis gene expression (Figure S2B). Regarding auxin biosynthesis, expression of *TRYPTOPHAN AMINOTRANSFERASE OF ARABIDOPSIS 1* (*TAA1*) and *YUCCA 6* (*YUC6*) was repressed in the leaf tip while *YUC2*, *YUC5*, *YUC8*, and *YUC9* expression was induced. In contrast, *YUC3* expression was specifically induced in the petiole. Investigating GA biosynthesis, we found tissue-specific induction of *GA20 OXIDASE 1* (*GA20OX1*) and *GA20OX2* in the petiole and *GA20OX3* in the leaf tip. Downstream of GA20OX proteins, *GA3 OXIDASE 1* (*GA3OX1*) was induced in all tissues. Regarding ABA biosynthesis, we found induction of *NCED3* in the leaf tip while *NCED5* was induced in the petiole. Furthermore, we observed transcriptional regulation of genes involved in the biosynthesis of BR, ethylene, and other hormones (Figure S2B).

**Figure 2:**
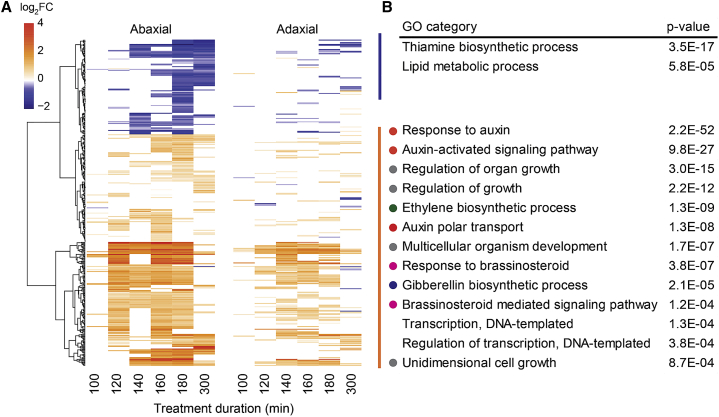
Neighbor detection at the leaf tip induces unique abaxial and adaxial transcriptomes (A) Clustered heatmap showing log_2_FC in FRtip compared with WL of genes showing a different FRtip response between the two petiole sides at the indicated time points (ANOVA interaction tissue ^∗^ treatment p < 0.001). (B) Separate GO analysis based on the clusters of upregulated (orange-red) and downregulated (blue) genes identified in (A). p values indicate the enrichment significance of the associated GO category. Colored circles represent the following defined major biological processes: red, auxin distribution and signaling; gray, cell and organ growth; green, ethylene biosynthesis; magenta, brassinosteroid signaling; blue, gibberellin biosynthesis. See also Data S2 and S3.

To better understand abaxial-adaxial transcript differences, we next identified genes showing differential response to FRtip between the two sides at 100–300 min of treatment (Figure 2). There were no genes with opposite regulation between the two sides, but we did observe consistently stronger transcript regulation in the abaxial petiole for both up- and downregulated DEGs (Figure 2A). The FRtip-upregulated genes in this subset showed enrichment for biological processes related to auxin, growth, GA, BR, and ethylene (Figure 2B). As transcript regulation is strongest in the abaxial petiole in this comparison, this suggests that these processes are preferentially activated abaxially. In this analysis, the transcripts with the lowest p values included many *SAUR*s and other auxin-induced genes as well as *GA20OX1* and *GA20OX2* (Data S2). Abaxial-adaxial transcriptional differences were also found in WL and included many photosynthesis-associated genes (Data S3).

### Neighbor detection in the leaf tip leads to directed auxin transport toward the abaxial petiole

**Figure 3:**
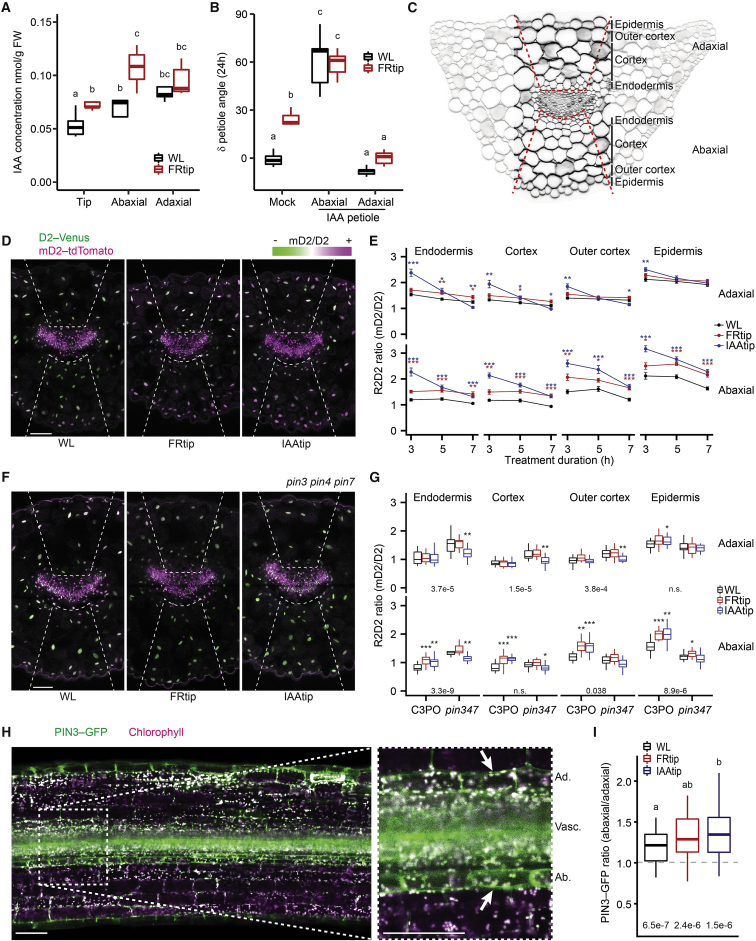
Leaf-tip-derived auxin is directed toward the abaxial petiole via PIN transporters (A) Free IAA concentration (nmol/g FW) in the leaf tip and abaxial-adaxial split petiole after 5-h light treatment (n = 5 biological replicates from 20 plants each, different letters indicate significant differences, Fisher’s LSD, p < 0.05). (B) Petiole angle change after 24-h light treatment combined with 30 μM IAA or mock application to the petiole (n = 7, different letters indicate significant differences, Tukey HSD p < 0.05). (C) Petiole base cross-section indicating cell layers and petiole region that was used to quantify fluorescence in (D)–(G) and other figures. (D and E) Representative images after 5 h (D) and quantification at indicated time points (E) of the R2D2 ratio in the C3PO petiole base. Plants were treated with mock, FRtip, or IAAtip (n ≥ 11, colored asterisks represent significant treatment effect compared with WL, ^∗^p < 0.05, ^∗∗^p < 0.01, ^∗∗∗^p < 0.001, two-sided t test, data represent mean ± SEM). (F and G) Representative images of *pin3 pin4 pin7* C3PO (F) and quantification of the R2D2 ratio in the C3PO and *pin3 pin4 pin7* C3PO (*pin347*) petiole base (G). Plants were treated for 7 h with mock, FRtip or IAAtip (n ≥ 15, asterisks indicate significant treatment effect compared with WL, ^∗^p < 0.05, ^∗∗^p < 0.01, ^∗∗∗^p < 0.001, two-sided t test. Inset values represent p value for genotype difference in WL calculated per cell layer, two-sided t test.). (H) Representative overview image and close up around the petiole base vasculature (the zone that was also used for C3PO imaging) of *PIN3*_*pro*_*:PIN3-GFP* in a longitudinal petiole cross-section. Ad., adaxial endodermis; Vasc., vasculature; Ab., abaxial endodermis. Arrows indicate the endodermal cells where PIN3-GFP intensity in the membranes was quantified for (I). (I) Ratio of PIN3-GFP intensity in the abaxial/adaxial endodermis after 2.5–4 h in WL, FRtip, or IAAtip. Variation in treatment time was similar between treatments (n = 46, WL; 28, FRtip; 30, IAAtip; different letters indicate significant differences, Tukey HSD p < 0.05). Inset values represent p value for difference from ratio 1, one-sample t tests. Scale bars in microscopy images represent 100 μm; dashed lines in (D) and (F) indicate the abaxial and adaxial regions where nuclear fluorescence was quantified. In FRtip, the R/FR is locally reduced from 2.0 to 0.05. See also Figures S3 and S4.

The enrichment for FRtip-induced auxin signaling in the transcriptome prompted us to quantify free levels of the auxin indole-3-acetic acid (IAA) in the three leaf sections. We found increased IAA concentrations in the leaf tip and abaxial petiole, but not in the adaxial petiole upon FRtip exposure (Figure 3A). To study whether such differential auxin concentrations are required for petiole hyponasty, we exogenously applied IAA to the abaxial or adaxial petiole (Figure 3B). We found that abaxial IAA application induces strong hyponasty, regardless of R/FR, whereas adaxial IAA application inhibited the hyponastic response to FRtip. These observations indicate that an auxin gradient, either installed endogenously or through external application, is necessary for leaf movement.

To achieve further spatiotemporal resolution, we visualized auxin distribution using the newly constructed C3PO fluorescent auxin reporter. C3PO conveniently combines the previously described R2D2 auxin concentration reporter and the DR5v2 auxin response reporter^19^ into a single construct (*DR5v2:n3mTurquoise2 RPS5A*_*pro*_*:mD2-ntdTomato RPS5A*_*pro*_*:D2-n3Venus*) (Figure S3). We developed a method to image transverse cross-sections of fixated and cleared petioles and measured fluorescence in individual cell layers (Figures 3C and S3H). We found that the auxin concentration, as approximated by the mD2/D2 (R2D2) intensity ratio, increased in all abaxial cell layers within 3 h of FRtip and remained higher than WL throughout the measured 7 h interval, while there was little increase on the adaxial side (Figures 3D and 3E). When substituting FRtip with local IAA application on the leaf tip (IAAtip), we found increased R2D2 ratios after 3 h in both petiole sides. At later IAAtip treatment time points, the adaxial increase was lost and even changed into decreased R2D2 ratios in the adaxial endodermis and cortex, whereas the abaxial tissues continued to have an elevated R2D2 ratio.

### Auxin accumulation in the abaxial petiole via PINs

The petiole hyponasty response to FRtip requires intact auxin transport and is, therefore, reduced in the *pin3* single mutant and absent in the *pin3 pin4 pin7* triple mutant.^10^^,^^11^ Similarly, *pin3* and *pin3 pin4 pin7* mutants, respectively, showed reduced and absent hyponasty in response to IAAtip (Figure S4A). When we crossed C3PO to the *pin3 pin4 pin7* mutant background, we found that these mutations inhibited FRtip and IAAtip-induced abaxial R2D2 ratio increases (Figures 3F and 3G). In WL, the R2D2 ratio in *pin3 pin4 pin7* was relatively increased compared with wild type in the inner cell layers and reduced in the abaxial outer cortex and epidermis. We observed similar differences from wild type when regarding the auxin response, visualized by *DR5v2:mTurquoise2* (Figures S4B and S4C), implying that perturbed PIN function hampers auxin transport from the inner toward the outermost cell layers in the petiole. In contrast with the induction of the R2D2 ratio by FRtip and IAAtip in C3PO, there was no clear *DR5v2:mTurquoise2* intensity induction (Figures S4B and S4C). The lack of DR5v2 inducibility by IAAtip and FRtip likely indicates a poor sensitivity of this reporter in the petiole since we previously showed induction of auxin signaling using *DR5:Luciferase*,^11^ and our transcriptome analysis shows a pronounced auxin response upon FRtip (Figure 1). It is likely that activity of DR5v2 in the petiole is generally close to saturation, so that only large reductions in auxin signaling, such as following from the *pin3 pin4 pin7* triple mutations, affect the DR5v2 signal in petiole tissue.

Given the prominent effect of *pin* mutations on hyponasty (Figure S4A), the abaxial auxin accumulation in response to IAAtip and FRtip (Figure 3G), and the established regulation of PIN3 localization by supplemental FR in seedlings,^20^ we studied PIN3 localization and abundance in the petiole base using *PIN3*_*pro*_*:PIN3-GFP*. Since the fixation and clearing protocols used for the nuclear C3PO reporter did not work for PIN3-GFP, we used hand-made longitudinal sections in live tissue. In the petiole base endodermis, PIN3--GFP was significantly enriched on the abaxial side compared with the adaxial side and this asymmetry strengthens in IAAtip (Figures 3H and 3I). Taken together, this implies that PIN-dependent auxin transport directs tip-derived auxin to the abaxial petiole to stimulate abaxial cell elongation and petiole hyponasty upon neighbor detection in the leaf tip. Indeed, PIN3-driven auxin accumulation stimulates cell elongation responses to light, such as phototropic bending^21^ and unidirectional hypocotyl elongation in shade.^17^^,^^20^

### Auxin-mediated hyponasty acts via ARF and PIF transcription factors

**Figure 4:**
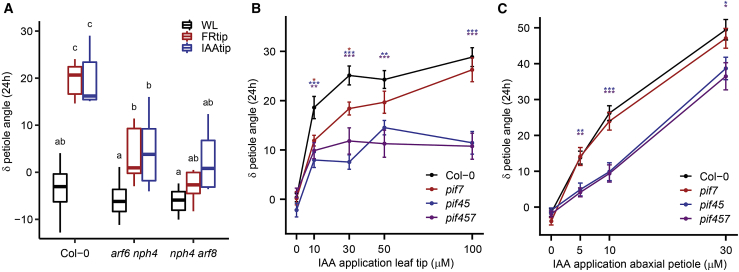
Leaf-tip-derived auxin stimulates petiole hyponasty through PIFs and ARFs (A) Petiole angle change after 24-h WL, FRtip, or IAAtip treatment in Col-0, *arf6 nph4*, and *nph4 arf8* (n = 7, different letters indicate significant differences, Tukey HSD p < 0.05). (B and C) Petiole angle change after 24 h in Col-0, *pif7*, *pif4 pif5* (*pif45*) and *pif4 pif5 pif7* (*pif457*) treated with different IAA concentrations or mock to the leaf tip (B) and abaxial petiole (C) (n = 14, colored asterisks represent significant genotype effect compared with Col-0, ^∗^p < 0.05, ^∗∗^p < 0.01, ^∗∗∗^p < 0.001, two-sided t test, data represent mean ± SEM). In FRtip, the R/FR is locally reduced from 2.0 to 0.05.

Upon arrival in target tissue, auxin can stimulate growth by activating target gene expression via AUXIN RESPONSE FACTOR (ARF) transcription factors.^22^ Mutant phenotyping revealed that higher order mutant combinations of *ARF6*, *ARF7* (*NON-PHOTOTROPIC HYPOCOTYL 4*, *NPH4*), and *ARF8*, which were previously described to collectively regulate hypocotyl elongation responses,^23^ reduce the hyponastic response to tip-derived auxin (Figure 4A). ARF6 is a member of the BAP/D module, in which the transcription factors BZR1, ARF6, and PIF4 stimulate cell growth by reinforcing each other’s activity while all being repressed by DELLAs.^24^ PIF4, PIF5, and PIF7 together regulate FR-induced hyponasty^11^ and mutation of *PIF4* and *PIF5* also reduced IAAtip-induced petiole hyponasty (Figure 4B). Loss of PIF7, in wild type or *pif4 pif5* background, however, had little to no effect on the responsiveness to IAAtip. We observed a similar pattern upon IAA application to the abaxial petiole (Figure 4C), confirming that the auxin response, and not auxin transport, is reduced in *pif4 pif5*, but not in *pif7*. Combined with our previous observation that FR-induced expression of *YUCCA* in the leaf tip is PIF7 dependent^11^ and the fact that PIF4 and PIF5 were previously shown to promote auxin sensitivity and response in hypocotyls,^15^^,^^25^ we conclude that PIF7 is required for YUCCA-mediated auxin biosynthesis in the leaf tip, while PIF4 and PIF5 promote the auxin response in the petiole.

### Gibberellin as a downstream target of auxin signaling

**Figure 5:**
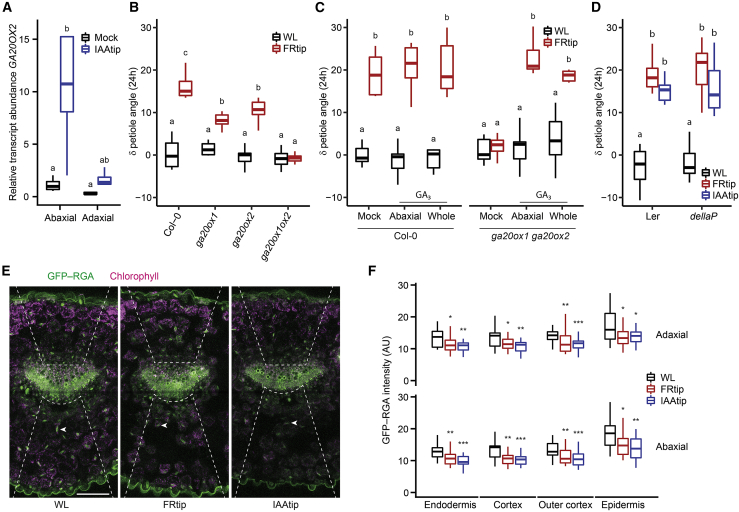
Gibberellin signaling facilitates the petiole hyponasty response to leaf tip-derived auxin (A) Relative *GA20OX2* transcript abundance in the abaxial and adaxial petiole after 2-h mock and IAAtip treatments, compared with the abaxial petiole in mock (n = 4 biological replicates from 8 plants each, different letters indicate significant differences, Tukey HSD p < 0.05). (B) Petiole angle change after 24 h light treatment in Col-0, *ga20ox1*, *ga20ox2*, and *ga20ox1 ga20ox2* (*ga20ox1ox2*) (n = 9, different letters indicate significant differences, Tukey HSD p < 0.05). (C) Petiole angle change after 24 h light treatment combined with 50 μM GA_3_ or mock application to the abaxial or whole (abaxial and adaxial) petiole in Col-0 and *ga20ox1 ga20ox2* (n = 7, different letters indicate significant differences, Tukey HSD p < 0.05). (D) Petiole angle change after 24-h WL, FRtip, or IAAtip treatment in L*er* and *dellaP* (n = 7, different letters indicate significant differences, Tukey HSD p < 0.05). (E and F) Representative images (E) and quantification (F) of *RGA*_*pro*_*:GFP-RGA* fluorescence in the petiole base. Plants were treated for 7 h with mock, FRtip, or IAAtip (n > 20, asterisks represent significant treatment effect compared with WL, ^∗^p < 0.05, ^∗∗^p < 0.01, ^∗∗∗^p < 0.001, two-sided t test). Scale bar in (E) represents 100 μm; dashed lines indicate the abaxial and adaxial regions where nuclear GFP signal was quantified, arrowheads point out an individual nucleus in the abaxial cortex in each image. In FRtip, the R/FR is locally reduced from 2.0 to 0.05. See also Figure S5 and Table S1.

The growth-repressing BAP/D module members, the DELLA proteins, are degraded through GA signaling.^26^ Our transcriptome analysis reveals enrichment for GA biosynthesis and signaling, specifically in the abaxial petiole (Figures 1F and 2), where expression of *GA20OX1* and *GA20OX2* was induced (Figure S2B). This seems to be a response to tip-derived auxin as similar asymmetric induction of *GA20OX2* was found in IAAtip (Figure 5A).

Mutant analysis revealed reduced hyponastic responses to FRtip in *ga20ox1* and *ga20ox2* mutants, and completely absent petiole hyponasty in *ga20ox1 ga20ox2* double mutants (Figure 5B). When we applied GA to either the abaxial petiole or both petiole sides, FRtip-induced hyponasty was restored in *ga20ox1 ga20ox2* (Figure 5C). Consistently, paclobutrazol (PAC) pre-treatment, which blocks GA biosynthesis, also inhibited FRtip-induced hyponasty that could be rescued by exogenous petiole GA application (Figure S5A). In addition, we observed petiole hyponasty when we applied GA to the leaf tip (GAtip) without additional FR in both wild type and *ga20ox1 ga20ox2* (Figure S5B). GAtip also rescued petiole elongation to wild-type levels in *ga20ox1 ga20ox2* (Figure S5C). Petiole hyponasty was not observed when GA was applied directly to the abaxial petiole (Figure 5C), suggesting that GAtip might generate another signal to induce hyponasty. As GA presence leads to DELLA degradation, DELLA proteins repress PIFs and PIFs facilitate auxin synthesis by enhancing *YUCCA* expression, we analyzed whether GAtip-induced auxin signaling. Using the previously employed *DR5:Luciferase* reporter,^11^ we found that GAtip indeed mildly induced auxin signaling in the leaf tip (Figure S5D). In addition, transcript levels of *YUC9*, but not *YUC2*, *YUC5*, or *YUC8* were induced by GAtip treatment in the leaf tip (Figure S5E) and *yuc2 yuc5 yuc8 yuc9* had a reduced hyponastic response to GAtip (Figure S5F). These data together suggest that GAtip-induced hyponasty could at least partially act via induction of auxin synthesis in the leaf tip.

Next, we tested the global (pentuple) DELLA knockout mutant *dellaP*. Although leaf angles were constitutively high in *dellaP*, FRtip and IAAtip still induced further petiole hyponasty, resulting in nearly vertical leaves (Figures 5D and S5G). When we studied DELLA abundance using the DELLA reporter *RGA*_*pro*_*:GFP-RGA* that was previously shown to be GA-sensitive in low R:FR, we observed clear RGA degradation in both petiole sides upon FRtip and IAAtip (Figures 5E and 5F), similar to the degradation caused by exogenous GA treatment (Figure S5H). These data together indicate that leaf-tip-derived auxin induces the expression of *GA20OX* GA synthesis genes in the petiole, presumably increasing GA levels required for auxin-induced hyponasty.

## Discussion

Here, we show that plants use directional auxin transport from the leaf tip toward the abaxial petiole to initiate petiole hyponasty upon neighbor detection in the leaf tip. Using transcriptome analyses, we reveal that phy signaling of FR light in the leaf tip induces a rapid auxin response in the abaxial petiole, that also stimulates expression of *GA20OX* GA biosynthesis genes (Figures 1, 2, and S3).

**Figure 6:**
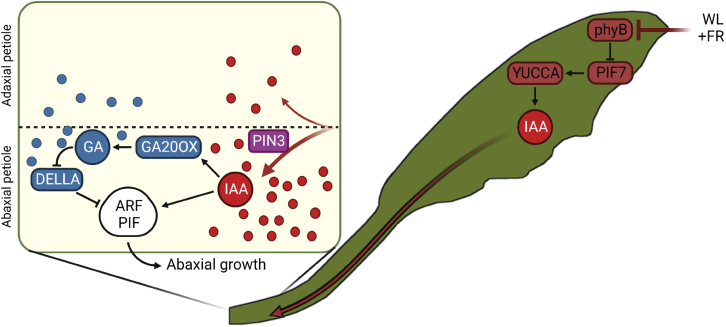
Proposed mechanism of how long-distance phytochrome signaling from tip to base orchestrates petiole hyponasty FR-enriched light reflected from neighbors is first detected at the outermost leaf tip. This induces local inactivation of phyB, followed by auxin synthesis via PIF7 and YUCCAs, of which gene expression is induced within 40 min of FRtip. Auxin is transported from the leaf tip to the petiole and directed toward the abaxial petiole by PINs. In the abaxial petiole, leaf-tip-derived auxin acts via ARFs and PIFs to promote local growth stimulation and likely stimulates gibberellin synthesis via *GA20OX* expression, leading to the breakdown of DELLAs in the petiole. DELLA inactivation would then release repression of the auxin-activated growth-promoting ARF-PIF transcription factor module. The asymmetric auxin distribution and signaling ensures that the cell growth is limited to the abaxial petiole which results in adaptive petiole hyponasty. Round shapes represent auxin (IAA, red) and gibberellin (GA, blue).

The directed auxin transport toward the abaxial petiole requires functional PIN auxin efflux proteins (Figures 3F, 3G, and S4), including PIN3. In WL control conditions PIN3 is more abundant in the abaxial than the adaxial petiole endodermis and this PIN3 asymmetry is enhanced in response to auxin application at the leaf tip (Figures 3H and 3I). The PIN3 asymmetry likely directs tip-derived auxin flow from the vasculature toward the abaxial petiole, thereby stimulating asymmetric growth and hyponasty (Figure 6). PIN4 and PIN7 localization dynamics may also contribute to directional auxin flow, potentially in other cell layers, as occurs in roots,^27^ but this was not investigated here. In seedlings, endodermal PIN3 asymmetry facilitates supplemental FR-light-induced hypocotyl elongation and blue light-mediated phototropism, both driven by cell expansion.^20^^,^^21^ Moreover, the petiole hyponastic response to elevated temperatures also involves PIN3 accumulation in the abaxial endodermis.^28^ Unlike these examples, which involve direct light or temperature treatment of the tissues where PIN3 asymmetries are installed, we show here that innate asymmetry helps respond to a remotely sensed FR signal. This innate PIN3 asymmetry may be caused by the standard levels of auxin itself under control conditions.^20^ Other putative factors that could orchestrate PIN3 asymmetry include signaling via leaf-polarity factors,^28^^,^^29^^,^^30^ asymmetric leaf and vasculature structure (Figure 3C), gravity,^31^ and even a light signaling gradient within the tissue.^32^

An earlier study on ethylene-induced hyponasty combined computational modeling and experiments to show that modest epidermal cell elongation in the proximal petiole, similar to what we observed here (Figure 1B), is more than sufficient to induce strong hyponasty.^12^ We, therefore, propose that auxin-mediated cell elongation in the abaxial proximal petiole promotes hyponasty.

As shown in Figures 3D–3G, auxin accumulates in all abaxial cell layers upon FR treatment, but whether it also actively regulates cell growth in all these cell layers remains unclear. Likewise, GA-dependent DELLA degradation also occurs in all these cell layers upon FR treatment (Figures 5E and 5F). In seedlings, it was shown that epidermal cell elongation is required for auxin-induced hypocotyl elongation.^33^ Similarly, inhibiting auxin signaling in the petiole epidermis represses low R/FR-induced hyponasty.^10^ We hypothesize that, as FRtip induces cell elongation in the abaxial petiole epidermis (Figure 1B), FRtip increases vascular auxin flow, which is directed to the abaxial petiole epidermis via asymmetrically localized endodermal PIN3. Although auxin may also promote cortex cell growth, the increased auxin signal in cortex cells might simply indicate that the cortex is a transit zone for auxin going from endodermis to epidermis.

Upregulation of *GA20OX* expression during shade and auxin-induced growth was previously shown.^34^^,^^35^ However, it was not known that these genes are also responsive to remote FR or auxin signaling. We observed that tip-derived auxin stimulates expression of the GA biosynthesis genes *GA20OX1* and *GA20OX2* in the growing abaxial petiole (Figures 5A and S2B). The resulting elevated GA levels would then enhance degradation of the DELLA protein RGA (Figures 5E and 5F), preventing DELLA-mediated inhibition of various growth-promoting transcription factors, including PIFs.^36^^,^^37^ In contrast to the specifically abaxial auxin accumulation and *GA20OX* expression, RGA degradation occurred on both petiole sides in FRtip (Figures 5E and 5F), suggesting abaxial-adaxial GA transport resulting in non-differential GA signaling in the petiole. When we applied GA to both petiole sides in GA-deficient *ga20ox1 ga20ox2* mutants, we found that the hyponastic response to FRtip was similarly rescued compared with when GA was applied only abaxially, and that GA application to the petiole in WL did not affect petiole angles (Figure 5C). We therefore propose that GA abundance and subsequent DELLA degradation in the petiole are required to allow for petiole cell growth, while abaxial auxin accumulation provides the directional cue that ensures differential petiole growth resulting in adaptive petiole hyponasty (Figure 6).

In contrast with *GA20OX1* and *GA20OX2*, *GA20OX3* was specifically induced in the leaf tip by FRtip. Our proposed mechanism for auxin-induced GA biosynthesis in the petiole via *GA20OX1* and *GA20OX2* does not exclude possible GA transport from the leaf tip toward the petiole to enhance petiole hyponasty. Indeed, when we applied GAtip in WL, this induced petiole hyponasty in both wild type and *ga20ox1 ga20ox2* (Figure S5B). As GA treatment of either the abaxial or the whole petiole fails to stimulate petiole hyponasty in WL (Figure 5C), we hypothesize that GAtip treatment locally degrades DELLAs, enhancing some PIF activity and auxin biosynthesis in the leaf tip. Indeed, we observed that GAtip stimulates auxin signaling and *YUC9* gene expression in the leaf tip (Figures S5D and S5E) and that GAtip-induced hyponasty was reduced in the *yuc2 yuc5 yuc8 yuc9* quadruple auxin biosynthesis mutant (Figure S5F). As *ga20ox1 ga20ox2* requires GA supplementation to the petiole to show petiole hyponasty in FRtip (Figure 5C), this suggests that exogenous GAtip is also transported toward the petiole. Combined tip-to-base transport of GA and GA-induced auxin would then allow for GAtip-induced hyponasty in *ga20ox1 ga20ox2*.

Using mutant analysis, we showed that ARF6, ARF7, and ARF8 as well as PIF4 and PIF5 are required for the petiole hyponasty response to tip-derived auxin (Figures 4A–4C). In addition, we found transcriptional activation of BR signaling in the petiole (Figures 1F and 2). This indicates that BAP/D module members are involved in auxin-mediated petiole hyponasty (Figure 6). Whether the specific members and interactions in the BAP/D module are the same in adult petioles as in hypocotyls^24^ remains to be studied.

Spatial separation of light signaling and shoot growth response were studied in seedlings in the past.^16^^,^^17^^,^^38^ However, the study system presented here provides an opportunity to study FR enrichment effects on distal, auxin-mediated growth without local light exposure of the responding organ. This will help further unravel the complex interactions between photoreceptors, the BAP/D module and other growth repressors and activators that plants use to optimize their growth to the environment.^39^ We conclude that upon neighbor detection, plants use carefully controlled long-distance auxin transport from the leaf tip to the abaxial petiole base to locally induce cell growth and adaptively raise their leaves in a process that requires GA biosynthesis and activity of PIF and ARF transcription factors.

## STAR★Methods

### Key resources table

**Table:** 

REAGENT or RESOURCE	SOURCE	IDENTIFIER
**Bacterial and virus strains**		

E.coli (DH5α)	N/A	N/A
A. tumefaciens (GV3101 pSOUP)	N/A	N/A

**Chemicals, peptides, and recombinant proteins**		

Indole-3-acetic acid	Duchefa	I0901
Gibberellic acid (GA_3_)	Duchefa	G0907
Paclobutrazol	Duchefa	P0922
Dental paste mixture	President	REF 60019949
SuperScript III Reverse Transcriptase	Thermo Fisher Scientific	18080093
Random hexamer primers	Thermo Fisher Scientific	N8080127
[^13^C_6_] Indole-3-acetic acid (^13^C-IAA) [3-[^13^C_6_] indolylacetic acid]	Oldchemim	Catalog number: 031 7333; CAS: 100849-36-3
D-Luciferin potassium salt	BioVision	7903
Calcofluor white	Sigma	F3543

**Critical commercial assays**		

RNeasy Mini Kit	QIAGEN	74104
RNase-Free DNase Set	QIAGEN	79254
SYBR Green Supermix	Bio-Rad	1725271

**Deposited data**		

RNA sequencing	GEO repository	https://www.ncbi.nlm.nih.gov/geo/query/acc.cgi, accession number GSE218944
RNA sequencing Shiny application	Shiny	https://kupersetal.shinyapps.io/Shiny/

**Experimental models: Organisms/strains**		

*A. thaliana*: wild type Col-0	N/A	N/A
*A. thaliana*: wild type L*er*	N/A	N/A
*A. thaliana*: *ga20ox1-3*	Rieu et al.^40^	N/A
*A. thaliana*: *ga20ox2-1*	Rieu et al.^40^	N/A
*A. thaliana*: *ga20ox1-3 ga20ox2-1*	Rieu et al.^40^	N/A
*A. thaliana*: *arf6-2 nph4-1*	Reed et al.^23^	N/A
*A. thaliana*: *nph4-1 arf8-3*	Reed et al.^23^	N/A
*A. thaliana*: *pif4-101 pif5-1*	Lorrain et al.^41^	N/A
*A. thaliana*: *pif7-1*	Leivar et al.^42^	N/A
*A. thaliana*: *pif4-101 pif5-1 pif7-1*	de Wit et al.^43^	N/A
*A. thaliana*: *pin3-3*	Friml et al.^44^	N/A
*A. thaliana*: *pin3-3 pin4 pin7*	Willige et al.^45^	N/A
*A. thaliana*: *pin3-3 PIN3*_*pro*_*:PIN3–GFP*	Žádníkova et al.^46^	N/A
*A. thaliana*: C3PO	This study	N/A
*A. thaliana*: *pin3 pin4 pin7* C3PO	This study	N/A
*A. thaliana*: *DR5:Luciferase*	Moreno-Risueno et al.^47^	N/A
*A. thaliana*: *yuc2 yuc5 yuc8 yuc9*	Kohnen et al.^17^	N/A
*A. thaliana*: *dellaP*	Feng et al.^36^	N/A
*A. thaliana*: *RGA*_*pro*_*:GFP–RGA*	Silverstone et al.^48^	N/A

**Oligonucleotides**		

All oligonucleotides	This study	Table S1

**Recombinant DNA**		

*GIIM*_*pro*_*/DR5v2:n3mTurquoise2 RPS5A*_*pro*_*:mD2–ntdTomato RPS5A*_*pro*_*:D2–n3Venus*	This study	N/A
*GIIK*_*pro*_*/DR5v2:n3mTurquoise2*	This study	N/A

**Software and algorithms**		

Icy Image analysis software	de Chaumont et al.^49^	https://icy.bioimageanalysis.org/
Fiji (ImageJ 1.53k)	Schindelin et al.^50^	https://fiji.sc/
R	R Core Team	https://www.r-project.org/
RNA-seq quality control, read annotation and Read Normalization	Utrecht Sequencing Facility	https://github.com/UMCUGenetics/RNASeq
DEG analysis	This study	https://github.com/plant-environment-signaling/Arabidopsis_hyponasty_transcriptomes
GO analysis	This study	https://github.com/plant-environment-signaling/Arabidopsis_hyponasty_transcriptomes
ggplot2	R package, open-source	RRID: SCR_014601
cowplot	R package, open-source	RRID: SCR_018081
pheatmap	R package, open-source	RRID: SCR_016418
zoo	R package, open-source	https://cran.r-project.org/web/packages/zoo/index.html
Shiny	R package, open-source	RRID: SCR_001626
Adobe Illustrator	Adobe	N/A
Zen microscopy software	Zeiss	N/A
ViiA 7 Software	Thermo Fisher Scientific	N/A

**Other**		

Magwell 96 well magnetic separator	Edge Biosystems	57624
Streptavadin Magnetic Beads	New England Biolabs	S1420S
0.45 μm Minisart SRP4 filter	Phenomenex	AF0-3102-52
Xevo TQ-S tandem quadrupole mass spectrometer	Waters	N/A
VT1000S Vibratome	Leica Biosystems	1404723512
Coverslip container	Lab-Tek	16210671
LSM880 Airyscan confocal microscope	Zeiss	N/A
LSM700 confocal microscope	Zeiss	N/A
SP5II confocal microscope	Leica	N/A
FR LEDs for FRtip	EPITEX	L730-06AU
FR LEDs for FRwhole	Philips	Green-Power 730 nm
Potting soil	Primasta	N/A

### Resource availability

#### Lead contact

Further information and request for resources and reagents should be directed to and will be fulfilled by the lead contact, Ronald Pierik (r.pierik@uu.nl).

#### Materials availability

All unique/stable reagents generated in this study are available from the lead contact with a completed Materials Transfer Agreement.

### Experimental model and subject details

#### Plant materials and growth conditions

All experiments in this study were performed using *Arabidopsis thaliana*. The used *Arabidopsis* lines are listed in the key resources table. For all experiments, 28 days old plants were selected based on homogeneous development and the presence of a ∼5 mm petiole on the 5^th^ youngest leaf which would be used in the experiment. For genotypes with reduced petiole length, a leaf of similar developmental stage was selected. Seeds were sown on Primasta soil or gas sterilized and sown on ½ MS 0.8 % Plant agar plates. Seeds were cold stratified for three days before transfer to short day white light (WL) conditions light/dark 9 h/15 h, 20 °C, 70 % humidity, 130-150 μmol m^-2^ s^-1^ PAR. Around eight days after germination, individual seedlings were transplanted to 70 mL round pots containing Primasta soil.

During C3PO generation, seeds were surface sterilized, sown on ½ MS 0.8 % Daishin agar and cold stratified for 2 days before transfer to long day climate room conditions light/dark 16 h/8 h, 22 °C.

### Method details

#### Construction of the C3PO auxin reporter

The C3PO construct (*GIIM*_*pro*_*/DR5v2:n3mTurquoise2 RPS5A*_*pro*_*:mD2–ntdTomato RPS5A*_*pro*_*:D2–n3Venus*) was generated via inserting *DR5v2:n3mTurquoise2* into R2D2.^19^ *n3mTurquoise2* was generated by sequentially cloning the following three constructs, that were generated via PCR from plasmid template *pmTurquoise2-C1100*, into *GIIK*_*pro*_*/LIC_SwaI-LIC_HpaIv2-tNOS*: *mTurquoise2* coding sequence (CDS) with a stop codon, *mTurquoise2* CDS without stop codon and *NLS–mTurquoise2* without stop codon. The *n3mTurquoise2-tNOS* cassette was then excised via BamHI-XbaI double-digestion and inserted via conventional cloning into *GIIK*_*pro*_*/DR5v2:ntdTomato-tNOS*, after the *ntdTomato-tNOS* cassette had first been removed via BamHI-XbaI double-digestion, to generate *GIIK*_*pro*_*/DR5v2:n3mTurquoise2-tNOS*. An AscI restriction site was inserted into XbaI-digested *GIIK*_*pro*_*/DR5v2:n3mTurquoise2-tNOS* via conventional cloning before ligating *DR5v2:n3mTurquoise2-tNOS*, that was excised by Bsp120I-AscI double-digestion, with Bsp120I-AscI double-digested *GIIM*_*pro*_*/RPS5A*_*pro*_*:mD2–ntdTomato RPS5A*_*pro*_*:D2–n3Venus* to generate *GIIM*_*pro*_*/DR5v2:n3mTurquoise2 RPS5A*_*pro*_*:mD2–ntdTomato RPS5A*_*pro*_*:D2–n3Venus* that we named C3PO. C3PO was then introduced into *Arabidopsis* via floral dip and selected using methotrexate. *pin3 pin4 pin7* C3PO was generated by crossing C3PO to *pin3-3 pin4 pin7*. Primer sequences used for cloning are shown in Table S1.

#### Light and pharmacological treatments

For FRtip light treatment, WL was supplemented with FR using EPITEX L730-06AU FR LEDs. These FR LEDs had peak emission at 730 nm and locally reduced R/FR from ∼2.0 in WL to ∼0.05 in FRtip. For FR treatment to the whole plant (FRwhole), WL was supplemented with FR using Philips Green-Power LEDs that had peak emission at 730 nm and reduced R/FR from 2.0 to 0.05. For pharmacological treatments at the leaf tip, 5 μL solution was pipetted onto the leaf tip. Except for the IAA concentration series in Figures 4B and S4A, 30 μM IAA was provided for IAAtip treatments. Pharmacological solutions and mocks for leaf tip application contained DMSO for IAA (0.03-0.1 %) or EtOH for GA_3_ (0.05 %) as well as Tween-20 (0.1 %). For hormone application to the petiole, concentrated stocks were diluted in lanolin (95-97 % lanolin, 0.01-0.03 % DMSO for IAA, 0.05 % EtOH for GA_3_). The lanolin containing solutions were carefully applied to the petiole using a tooth pick. When hormones were applied to one side of the petiole, a mock solution was applied to the other side. Paclobutrazol (PAC) treatment was done ten and five days before the experiment started. On both days, 20 mL 100 μM PAC or mock (0.3 % EtOH) was provided to the soil of each individual pot. All experiments were started at 10:00 (ZT2).

#### Epidermal imprints and cell size measurements

Leaf material for epidermal imprints was harvested after 24 hours treatment. Dissected petioles were gently pressed into dental paste mixture to produce a leaf mold. After a few minutes of drying, a thin layer of transparent nail polish was applied onto the partially hardened dental paste before application of a second layer of dental paste on the adaxial side of the petiole. After solidification, the petiole sample was removed from the dental paste and a thin layer of transparent nail polish was brushed onto the imprint. The nail polish film was mounted on a microscopy slide and imaged on a brightfield microscope at 40x magnification. Images were digitally stitched together and abaxial and adaxial cell lengths were measured along the petiole in ICY software.^49^ Data was smoothened with a rolling average combining cell length data from up to 5 x-axis positions, depending on whether neighbouring datapoints were available (using the zoo package in R).

#### qRT-PCR and RNA-sequencing

For gene expression experiments, leaf tip and petiole material was harvested and snap frozen in liquid nitrogen and stored at -80 °C until further processing. The number of plants per replicate and number of replicates used in qRT-PCR experiments are indicated in the figure legends. RNA for qRT-PCR was isolated using the Qiagen RNeasy kit with on-column DNase treatment. cDNA was synthesized using SuperScript III Reverse Transcriptase and random hexamer primers (Thermo Fisher). qRT-PCR was performed on the ViiA7 platform (Thermo Fisher) in 384-well plates using a 5 μL total volume containing SYBR Green (Bio-Rad). Transcript abundance was compared to housekeeping genes *PEX4* and *RHIP1* and made relative to the abundance in a designated control condition (indicated in figure legends). Primer sequences used for qRT-PCR are shown in Table S1. For RNA-sequencing, we harvested material from 13 leaves per sample, for a total of four biological replicates. Poly-A mRNA was isolated and used for the preparation of barcoded cDNA libraries according to the BrAD-seq protocol.^51^ Libraries were sequenced on an Illumina NextSeq 500 platform at 1^∗^75bp read length yielding around 13 million reads per sample.

#### RNA-sequencing data analysis

After quality control, reads were annotated to the TAIR10 genome and read counts were normalized using DESeq2 (https://github.com/UMCUGenetics/RNASeq, https://github.com/UMCUGenetics/RNASeq#differential-expression-analysis). Genes that had an average of less than 1 annotated read per sample were removed. For the remaining 19663 genes, we calculated the mean read count as well as log_2_FC and p-value between treatments. Treatment-induced differentially expressed genes (DEGs) were identified per timepoint and per tissue when p < 0.01 and log_2_FC > 0.3 / < -0.3. For Figure S2, a log_2_FC cut-off of > 1 / < -1 was used (https://github.com/plant-environment-signaling/Arabidopsis_hyponasty_transcriptomes). For Figure 2, we used an ANOVA approach to find genes with a significant (p < 0.001) two-way interaction Treatment^∗^Tissue between the two petiole halves at timepoints 100 - 300 minutes. Principal coordinate analysis was performed on log_2_ transformed relative transcript abundance. Gene ontology (GO) enrichment analyses were performed using the hypergeometric test available in R (https://github.com/plant-environment-signaling/Arabidopsis_hyponasty_transcriptomes). GO terms are only shown when highly significantly enriched in one sample (-log_10_(q-value) > 25) or consistently significantly enriched in five or more samples (-log_10_(q-value) > 5).

#### IAA extraction and quantification by liquid chromatography-tandem mass spectrometry

For the extraction of IAA from *A. thaliana* petioles, ∼40 mg of snap-frozen leaf material was used per sample. Tissue was ground to a fine powder at -80°C using 3-mm stainless steel beads at 50 Hz for 2^∗^30 seconds in a tissue lyser. Ground samples were extracted with 1 mL of cold methanol containing [phenyl ^13^C_6_]-IAA (0.1 nmol/mL) as an internal standard as previously described.^52^ Samples were filtered through a 0.45 μm Minisart SRP4 filter and measured on the same day. IAA was analyzed on a Waters Xevo TQs tandem quadrupole mass spectrometer as previously described.^53^^,^^54^

#### DR5:Luciferase analysis

For *DR5:Luciferase* analysis, the treated leaves were dissected from the plant and sprayed with 2 mM D-luciferin potassium salt in 0.1 % Triton X-100. A Bio-Rad ChemiDoc with a 25-30 minute exposure time was used to image luciferase luminescence.

#### Confocal microscopy

For confocal microscopy in transverse petiole cross-sections leaves were harvested into 24-well plates containing 4 % paraformaldehyde in PBS (pH 6.8) with Tween-20 (0.05 %). After vacuum incubation for one hour, leaves were washed three times for two minutes in PBS and stored for up to 24 h in PBS. Next, leaves were dried and placed in an Eppendorf tube containing warm agarose (3.5 %) and transferred to ice to solidify the agarose. Solid agarose plugs were sectioned to 250 μm slices using a Leica VT1000S vibratome. The first two slices from the petiole base (∼0-500 μm) were discarded, and the next two (∼500-1000 μm) were moved to 24-well plates containing ClearSee medium^55^ and incubated for at least 7 days before microscopy. For Figure 3C, after the initial clearing, ClearSee was supplemented with Calcofluor white (0.01 %, 5 h), and rinsed afterwards with ClearSee. Longitudinal cross-sections for PIN3–GFP were made by hand, without prior fixation or clearing. Samples were directly placed with the cut edge onto a coverslip container (Lab-Tek) and immediately imaged. Sample drying was prevented by adding wet filter paper around the sample and covering the combination with a coverslip. Imaging in these longitudinal cross-sections took place in the same basal region of the petiole as was used for transverse imaging (∼500-1000 μm from the petiole base).

Confocal microscopy was largely performed on a Zeiss LSM880 system using a 25x glycerol objective. For C3PO we used the following lasers and filters; mTurquoise2 – 458 nm laser, 467-500 nm filter, Venus – 514 nm laser, 525-550 nm filter, tdTomato – 561 nm laser, 571-629 nm filter. For PIN3–GFP we used; GFP – 488 nm laser, 501-548 nm filter, chlorophyll – 561 nm laser, 651-704 nm filter. For GFP–RGA we used the following lasers and filters in Figure 5E and 5F; GFP – 488 nm laser, 510-525 nm filter, chlorophyll – 561 nm laser, 641-691 nm filter. For Figure S5H we used a Zeiss LSM700 system using a 20x air objective with the following lasers and filters; GFP – 488 nm laser, 490-555 nm filter, chlorophyll – 555 nm laser, 560 – 1000 nm filter.

For the development of C3PO, confocal microscopy on roots was performed on a Leica SP5II system using a 20x water-immersion objective with the following laser and filters; mTurquoise2 – 458 nm laser, 468-495 nm filter, Venus – 514 nm laser, 524-540 nm filter, tdTomato – 561 nm laser, 571-630 nm filter.

### Quantification and statistical analysis

#### Image quantification

For phenotyping experiments, petiole angle before treatment and after 24 hours was determined in Fiji^50^ using side photos. Measurements of signal intensity for luciferase and fluorescence microscopy were performed in Icy bioimage analysis software.^49^ Luciferase intensity was derived by measuring mean pixel intensity in a region of the leaf tip margin that was kept constant in size between samples. For quantification of nuclear fluorescent protein intensity, Z-stacks were generated and combined into maximum intensity projections and mean fluorescence intensity was measured per individual cell. For PIN3–GFP, mean fluorescence intensity was measured on all sides of the visible endodermal cells in a single representative Z-layer. Icy was also used to select representative microscopy images and adjust brightness and contrast for improved clarity. Image adjustments were performed identically between treatments.

#### Statistical analyses and data visualisation

Specific details on statistical analyses and sample sizes can be found in the figure legends. In multi-comparison analyses, we performed multi-factorial ANOVA with Tukey’s HSD post hoc correction. Elsewhere, we used two-sided t-test with p < 0.05 cut-off. Graphs and heatmaps were prepared in R using the packages pheatmap, ggplot2 and cowplot and finetuned in Adobe Illustrator. The schematic model of signaling in Figure 6 was made in BioRender.

### Additional resources

We generated a Shiny application that can be used to easily determine the expression pattern of a gene of interest in our dataset using its unique Arabidopsis Gene Identifier (AGI): https://kupersetal.shinyapps.io/Shiny/.

## Data Availability

• RNA-seq data generated in the current study have been deposited at GEO: GSE218944 and are publicly available as of the date of publication. • All original code has been deposited at [https://github.com/plant-environment-signaling/Arabidopsis_hyponasty_transcriptomes] and is publicly available as of the date of publication. DOIs are listed in the key resources table. • Any additional information required to reanalyze the data reported in this paper is available from the lead contact upon request.

## Supplemental information

Document S1. Figures S1–S5 and Table S1

Data S1. Gene expression overview in all conditions and differential expression of genes between light treatments, related to Figure 1 Dataset provides normalized read counts per condition, as well as log2FC and p value between WL and FRtip, calculated per time point and tissue. (A) Leaf tip 40 min time point. (B) Leaf tip 60 min time point. (C) Leaf tip 80 min time point. (D) Leaf tip 100 min time point. (E) Leaf tip 120 min time point. (F) Leaf tip 140 min time point. (G) Leaf tip 160 min time point. (H) Leaf tip 180 min time point. (I) Leaf tip 300 min time point. (J) Abaxial petiole 60 min time point. (K) Abaxial petiole 80 min time point. (L) Abaxial petiole 100 min time point. (M) Abaxial petiole 120 min time point. (N) Abaxial petiole 140 min time point. (O) Abaxial petiole 160 min time point. (P) Abaxial petiole 180 min time point. (Q) Abaxial petiole 300 min time point. (R) Abaxial petiole 60 min time point. (S) Abaxial petiole 80 min time point. (T) Abaxial petiole 100 min time point. (U) Abaxial petiole 120 min time point. (V) Abaxial petiole 140 min time point. (W) Abaxial petiole 160 min time point. (X) Abaxial petiole 180 min time point. (Y) Abaxial petiole 300 min time point.

Data S2. Gene list showing FRtip-induced expression differences between the abaxial and adaxial petiole, related to Figure 2 Dataset provides mean normalized read counts from 100 to 300 min in the abaxial and adaxial petiole in both light treatments. In addition, this dataset shows the main and interaction effects for time point, tissue and treatment of an ANOVA analysis performed to identify genes with differential FRtip-induced regulation between the abaxial and adaxial petiole at 100–300 min of treatment.

Data S3. Gene list showing differential expression between the abaxial and adaxial petiole in WL, related to Figure 2 Dataset shows the main and interaction effects for time point and tissue of an ANOVA analysis performed to identify genes with differential gene expression between the abaxial and adaxial petiole in WL control conditions.

Document S2. Article plus supplemental information
