# Supplementary material for: Local light signaling at the leaf tip drives remote differential petiole growth through auxin-gibberellin dynamics
Source: Curr Biol. 2023 Jan 9;33(1):75–85.e5. doi: 10.1016/j.cub.2022.11.045 (PMC9839380; doi:10.1016/j.cub.2022.11.045)
Supplement: Document S1. Figures S1–S5 and Table S1 [file mmc1.pdf]

Current Biology, Volume *33*

Supplemental Information

Local light signaling at the leaf tip drives remote differential petiole growth through auxin-gibberellin dynamics

Jesse J. Küpers, Basten L. Snoek, Lisa Oskam, Chrysoula K. Pantazopoulou, Sanne E.A. Matton, Emilie Reinen, Che-Yang Liao, Eline D.C. Eggermont, Harold Weekamp, Muthanna Biddanda-Devaiah, Wouter Kohlen, Dolf Weijers, and Ronald Pierik

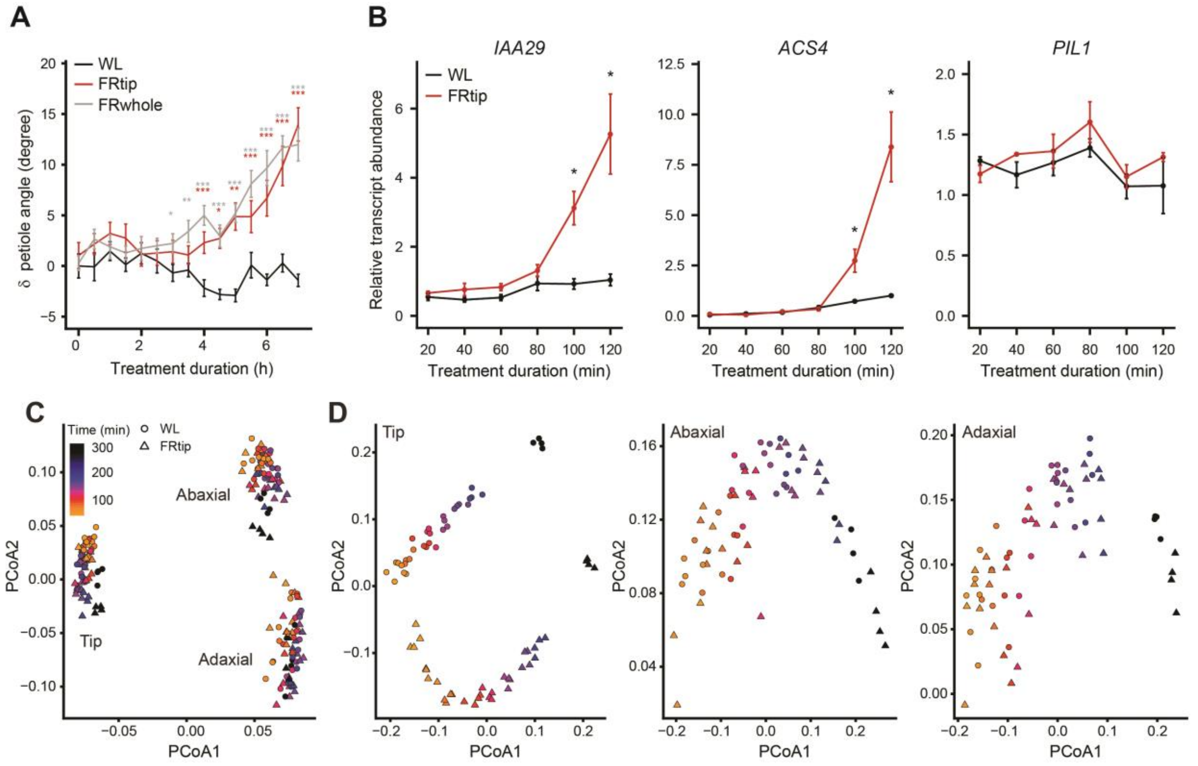

Figure S1. Design criteria and principal coordinate analysis of RNA-sequencing experiment, Related to Figure 1
(A) Petiole angle dynamics during the first 7 h of WL, FRtip and FR treatment to the whole plant (FRwhole). Petiole angle change is calculated relative to WL 0h treatment. (n = 10, coloured asterisks represent significant treatment effect compared to WL, *: $p < 0.05$, **: $p < 0.01$, ***: $p < 0.001$, two-sided t-test per timepoint, data represent mean ± SEM). (B) Relative transcript abundance in the petiole base of the auxin response marker genes *IAA29* and *ACS4* and the shade marker gene *PIL1* during the first 2h of WL and FRtip. Transcript abundance is calculated relative to 120 min WL. (n = 2 (20 & 40 minutes), n = 4 (60 – 120 minutes), material harvested from 7 plants per sample, *: $p < 0.05$, two-sided t-test per timepoint, data represent mean ± SEM). (C & D) Principal coordinate analysis on variation of DEseq2-normalized read count abundance on all samples (C) and separately per tissue (D). Each datapoint represents an individual sample from the leaf tip, abaxial or adaxial petiole and exposed to either WL or WL + FRtip, for 40 to 300 minutes. In FRtip treatment, the R/FR is locally reduced from 2.0 to 0.05. In FRwhole treatment, the R/FR is reduced from 2.0 to 0.05. See also Table S1.

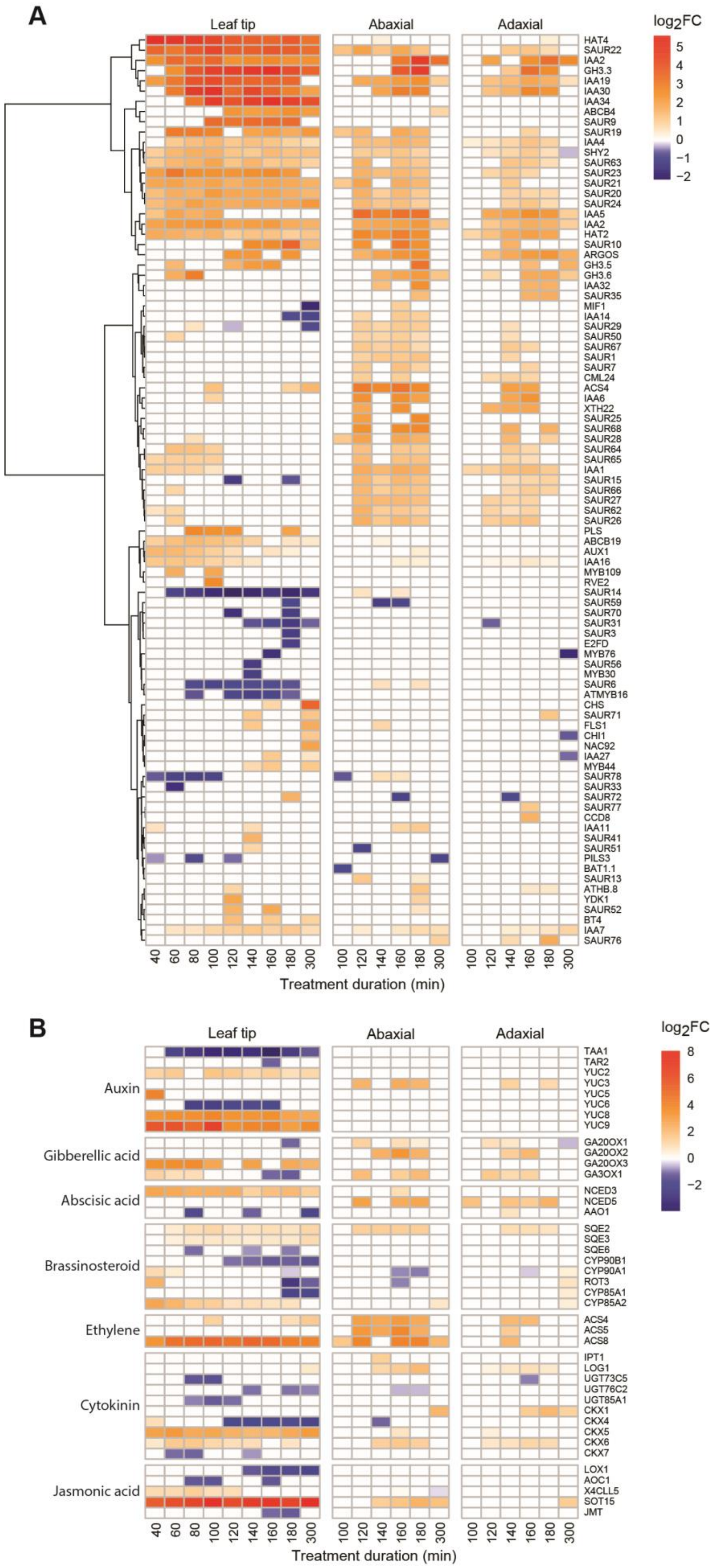
A
Leaf tip
Abaxial
Adaxial
log2FC
5
4
3
2
1
0
−1
−2
HAT4
SAUR22
IAA2
GH3.3
IAA19
IAA30
IAA34
ABCB4
SAUR9
SAUR19
IAA4
SHY2
SAUR63
SAUR23
SAUR21
SAUR20
SAUR24
IAA5
IAA2
HAT2
SAUR10
ARGOS
GH3.5
GH3.6
IAA32
SAUR35
MIF1
IAA14
SAUR29
SAUR50
SAUR67
SAUR1
SAUR7
CML24
ACS4
IAA6
XTH22
SAUR25
SAUR68
SAUR28
SAUR64
SAUR65
IAA1
SAUR15
SAUR66
SAUR27
SAUR62
SAUR26
PLS
ABCB19
AUX1
IAA16
MYB109
RVE2
SAUR14
SAUR59
SAUR70
SAUR31
SAUR3
E2FD
MYB76
SAUR56
MYB30
SAUR6
ATMYB16
CHS
SAUR71
FLS1
CHI1
NAC92
IAA27
MYB44
SAUR78
SAUR33
SAUR72
SAUR77
CCD8
IAA11
SAUR41
SAUR51
PILS3
BAT1.1
SAUR13
ATHB.8
YDK1
SAUR52
BT4
IAA7
SAUR76
40 60 80 100 120 140 160 180 300
100 120 140 160 180 300
100 120 140 160 180 300
Treatment duration (min)
B
Leaf tip
Abaxial
Adaxial
log2FC
8
6
4
2
0
−2
Auxin
TAA1
TAR2
YUC2
YUC3
YUC5
YUC6
YUC8
YUC9
Gibberellic acid
GA20OX1
GA20OX2
GA20OX3
GA3OX1
Abscisic acid
NCED3
NCED5
AAO1
Brassinosteroid
SQE2
SQE3
SQE6
CYP90B1
CYP90A1
ROT3
CYP85A1
CYP85A2
Ethylene
ACS4
ACS5
ACS8
Cytokinin
IPT1
LOG1
UGT73C5
UGT76C2
UGT85A1
CKX1
CKX4
CKX5
CKX6
CKX7
Jasmonic acid
LOX1
AOC1
X4CLL5
SOT15
JMT
40 60 80 100 120 140 160 180 300
100 120 140 160 180 300
100 120 140 160 180 300
Treatment duration (min)

Figure S2. Neighbour detection at the leaf tip induces tissue-specific auxin response and hormone biosynthesis, Related to Figure 1
(A) Clustered heatmap showing log_2FC in FRtip compared to WL of genes in the GO category “GO:0009733 response to auxin” calculated per timepoint and per tissue. (B) Heatmap showing log_2FC in FRtip compared to WL of genes involved in major hormone biosynthesis pathways calculated per timepoint and per tissue. In both heatmaps, only genes that have a significant treatment effect in at least one sample are shown ($p < 0.01$, $log_2FC > 1$ / < -1). In FRtip treatment, the R/FR is locally reduced from 2.0 to 0.05.

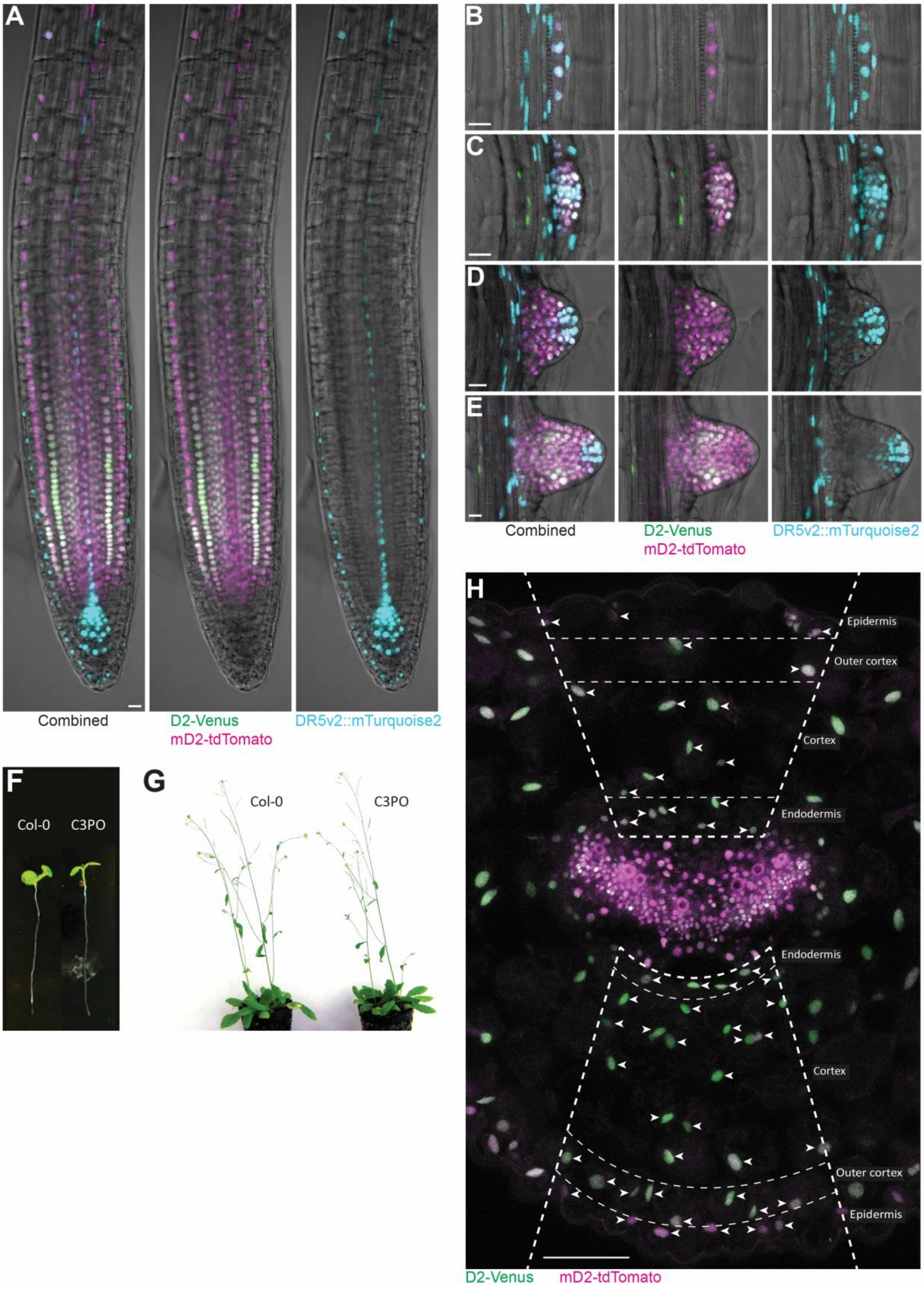

Figure S3. Description of C3PO fluorescence patterns and intensity quantification, Related to Figure 3
C3PO fluorescence patterns in the root tip (A), and lateral root primordium at stage I (B), stage IV (C), stage VI (D) and stage VIII (E) of 5 day old seedlings. (F & G) Plant phenotype of 6 day old seedling (F) and 35 day old flowering plant (G) of Col-0 and C3PO grown in 16 h/8 h light/dark conditions. (H) Close-up image of the transverse petiole cross-section of C3PO in WL (Figure 3D), showing D2-Venus and mD2-tdTomato fluorescence. Dashed lines indicate the region and cell types where C3PO fluorescence was imaged. Arrowheads indicate individual nuclei in which fluorescence intensity was quantified. Note that in the adaxial epidermis, stomatal fluorescence was not measured. Scale bars represent 10 µm in A – E and 100 µm in H. See also Table S1.

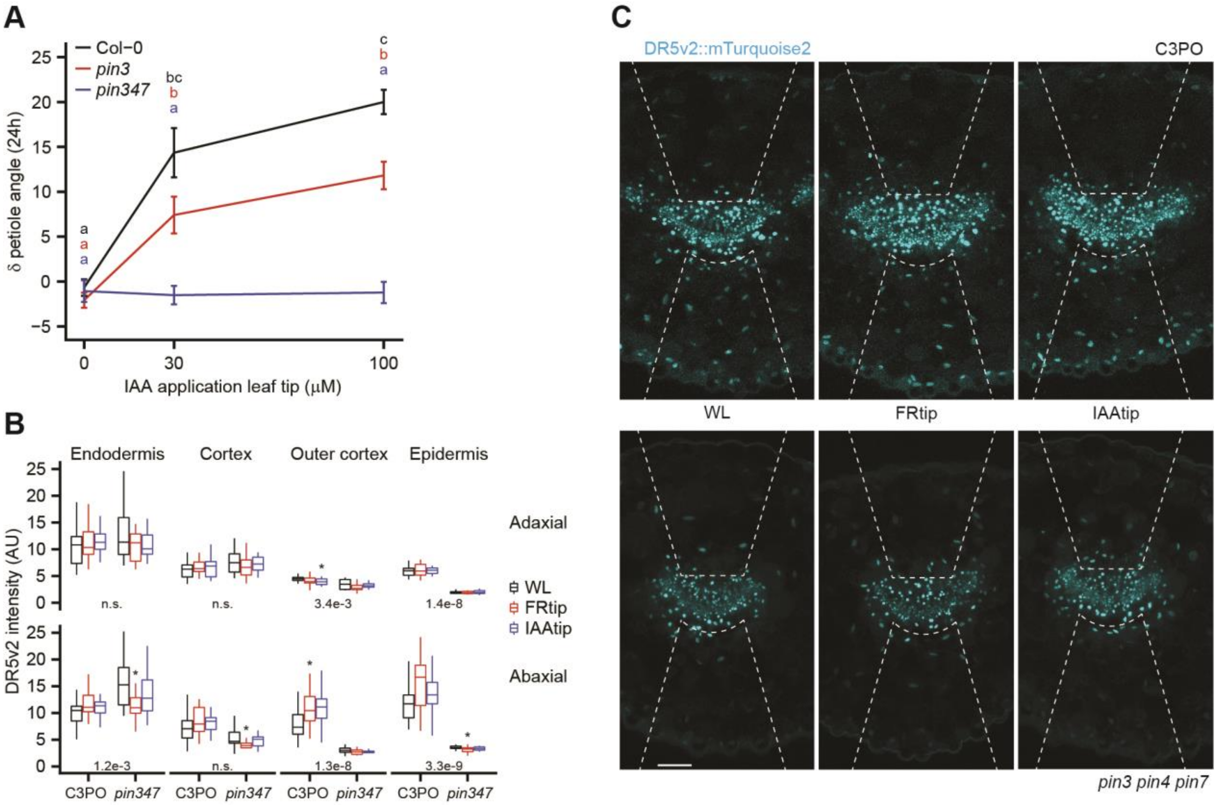

Figure S4. PIN auxin transporters are required for auxin signalling in the petiole and the hyponastic response to leaf tip-derived auxin, Related to Figure 3

(A) Petiole angle change after 24 h in Col-0, *pin3* and *pin3 pin4 pin7* (*pin347*) treated with different concentrations of IAA or mock to the leaf tip. (n = 10, different letters indicate significant differences with colours representing the corresponding genotype, Tukey HSD $p < 0.05$, data represent mean ± SEM). Representative images (B) and quantification (C) of DR5v2::mTurquoise2 fluorescence in the petiole base in C3PO and *pin3 pin4 pin7* C3PO. Plants were treated for 7 h with mock, FRtip or IAAtip. (n = > 15, asterisks indicate significant treatment effect compared to WL, *: $p < 0.05$, two-sided t-test. Inset values represent p-value for genotype difference in WL calculated per cell layer, two-sided t-test). Data from the same experiment as shown in Figures 3F and 3G. Scale bar represents 100 µm, dashed lines indicate the abaxial and adaxial regions where nuclear mTurquoise2 fluorescence was quantified. In FRtip treatment, the R/FR is locally reduced from 2.0 to 0.05.

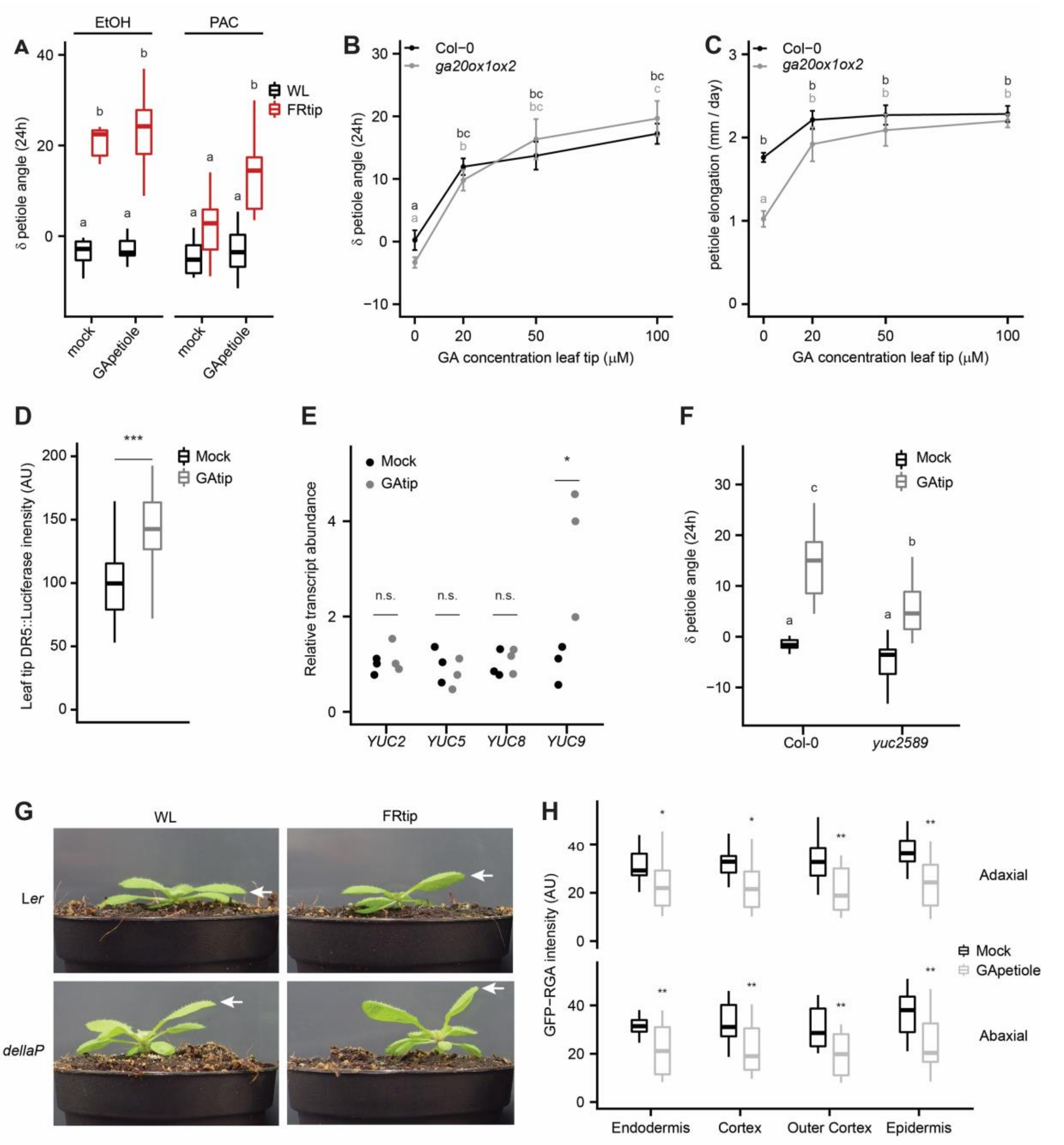

Figure S5. Gibberellin signalling stimulates petiole hyponasty, Related to Figure 5

(A) Petiole angle change after 24h in mock (EtOH) or PAC pre-treated Col-0 plants treated with WL or FRtip and mock or 50 μM GA_3 to the abaxial petiole. (n = 7, different letters indicate significant differences, Tukey HSD $p < 0.05$). (B & C) Petiole angle change (B) and petiole elongation (C) in Col-0 and *ga20ox1 ga20ox2* (*ga20ox1ox2*) after 24h mock or GA_3 treatment to the leaf tip in WL conditions. (n = 7, different letters indicate significant differences with colours representing the corresponding genotype, Tukey HSD $p < 0.05$, data represent mean ± SEM). (D) DR5::Luciferase signal intensity in the leaf tip after 7h mock or 50 μM GA_3 treatment to the leaf tip in WL conditions (n = 17, asterisks represent significant treatment effect ***: $p < 0.001$, two-sided t-test). (E) Relative transcript abundance normalised to mock of *YUC2*, *YUC5*, *YUC8* and *YUC9* in the leaf tip after 3.5h mock or 50 μM GA_3 treatment to the leaf tip in WL conditions (n = 3, asterisk represents significant treatment effect *: $p < 0.05$, two-sided t-test). (F) Petiole angle change in Col-0 and *yuc2 yuc5 yuc8 yuc9* (*yuc2589*) after 24h in mock or 50 μM GA_3 to the leaf tip. (n = 16, different letters indicate significant differences, Tukey HSD $p < 0.05$). (G) Representative images of L*er* and *dellaP* after 24h in WL and FRtip. The leaves of interest are indicated with the arrows. (H) Quantification of GFP-RGA fluorescence in the petiole base after 7h mock or 50 μM GA_3 treatment to the petiole. (n > 14, asterisks represent significant treatment effect compared to mock, *: $p < 0.05$, **: $p < 0.01$, two-sided t-test). In FRtip treatment, the R/FR is locally reduced from 2.0 to 0.05. See also Table S1.

Primer set	Forward	Reverse	Purpose
ACS4	GGAGCCACTTCCGCAAAC	GCTTGCTCGTAGGCTTCTTC	qPCR
IAA29	AAGATGGATGGTGTGGCAAT	GTCACCCTCTTTCCCTTGGA	qPCR
PIL1	AGACCACCTACGATGTTGCC	TAGCATTTGTGGTGGTGCAT	qPCR
GA20OX2	AGTAGCTTCACCGGCAGATT	ACGCCTAAACTTAAGCCCAGA	qPCR
YUC2	TGGAAACTCCGGGATGGAAGT	ACTTGAGCAGGCTCGTGGAT	qPCR
YUC5	AAGTGGTCTCTGGCGCATC	GAACAACTTTCTCCGCGTTT	qPCR
YUC8	TGCGGTTGGGTTTACGAGGAAAG	GCGTTTCGTGGGTTGTTTTG	qPCR
YUC9	AGTCCGGCGAGAAATTCAGA	AACCGAGCTTCTAACGACCA	qPCR
RHIP1	ATTGGTGTCGCTGCTAGTCT	TAAAGCCGTCCTCTCAAGCA	qPCR
PEX4	TGCAACCTCCTCAAGTTCGA	TGAGTCGCAGTTAAGAGGACT	qPCR
mTurqoise2 + STOP	TTTTGGATCCGGTGGTATGGT GAGCAAGGGCGAGGA	TTTTAGATCTTTACTTGTACA GCTCGTCCATGC	Cloning
mTurqoise2 non-STOP	TTTTGGATCCGGTGGTATGGT GAGCAAGGGCGAGGA	TTTTAGATCTCTTGTACAGCT CGTCCATGCC	Cloning
NLS-mTurqoise2 non-STOP	TTTTGGATCCCATGGCTCCAA AGAAGAAGAGAAAGGTCATG GTGAGCAAGGGCGAGGA	TTTTAGATCTCTTGTACAGCT CGTCCATGCC	Cloning
Additional AscI	CTAGATTAATTAAGACACAGG CGCGCCT	CTAGAGGCGCGCCTGTGTCT TAATTAAT	Cloning

Table S1. Primers used in this study, Related to Figures 5A, S1B, S3 and S5E
